# Extensive DNA Barcoding of Lepidoptera of Crete (Greece) Reveals Significant Taxonomic and Faunistic Gaps and Supports the First Comprehensive Checklist of the Island’s Fauna

**DOI:** 10.3390/insects16050438

**Published:** 2025-04-22

**Authors:** Peter Huemer, Kai Berggren, Leif Aarvik, Erwin Rennwald, Axel Hausmann, Andreas Segerer, Giorgia Staffoni, Aina Mærk Aspaas, Apostolos Trichas, Paul D. N. Hebert

**Affiliations:** 1 Naturwissenschaftliche Sammlungen, Sammlungs- und Forschungszentrum, Tiroler Landesmuseen Betriebsges.m.b.H., 6060 Hall in Tirol, Austria; 2 Independent Researcher, Bråvann Terrasse 21, 4624 Kristiansand, Norway; kberggr@online.no; 3 Natural History Museum, University of Oslo, 0318 Oslo, Norway; leif.aarvik@nhm.uio.no; 4 Independent Researcher, Mozartstr. 8, 76287 Rheinstetten, Germany; erwin@rennwald-biol.de; 5 Staatliche Naturwissenschaftliche Sammlungen Bayerns, Zoologische Staatssammlung München, Münchhausenstr. 21, 81247 München, Germany; hausmann.a@snsb.de (A.H.); segerer@snsb.de (A.S.); 6 Department of Biology, University of Florence, Via Madonna del Piano 6, 50019 Sesto Fiorentino, Italy; giorgia.staffoni@unifi.it; 7 Department of Natural History, NTNU University Museum, Norwegian University of Science and Technology, 7491 Trondheim, Norway; aina.aspaas@ntnu.no; 8 Natural History Museum of Crete, University of Crete, Knossos Av., 71409 Heraklion, Greece; atrichas@uoc.gr; 9 Centre for Biodiversity Genomics, University of Guelph, 150 Stone Road East, Guelph, ON N1G 2W1, Canada; phebert@uoguelph.ca

**Keywords:** checklist, Crete, DNA barcode, endemism, faunistics, Lepidoptera, taxonomy

## Abstract

Comprehensive genetic surveys of Lepidoptera are still largely lacking across most of the eastern Mediterranean. Consequently, there is a lack of modern, taxonomically validated checklists that meet current scientific standards. In this study, we analyze the butterfly and moth fauna of Crete (Greece) for the first time, based on 3110 DNA barcode sequences, primarily obtained from specimens based on our own sampling program. Building on these data, and incorporating previously published records from print sources and online forums, we establish the first comprehensive checklist of the island’s fauna. In total, the occurrence of 1230 species from 62 families is confirmed, with 724 of them genetically verified. Among them, 75 species appear to be island endemics. The checklist includes 125 newly recorded species for Crete, validated by DNA barcoding (with 36 also being new for Greece), along with 23 species confirmed solely through morphological study, and another 16 only documented by photographs. Conversely, 212 previously reported species had to be removed as likely invalid. Furthermore, 112 unidentified sequence clusters (BINs—Barcode Index Numbers) were documented, taxonomic uncertainties that will require future integrative resolution.

## Simple Summary

Despite their importance for monitoring and conservation, no recent inventories of butterflies and moths exist for countries in Southeastern Europe. Likewise, the Lepidoptera fauna of Crete have never been comprehensively documented, despite their biogeographical uniqueness and high degree of endemism. We address this gap through a critical analysis of the species inventory, coupled with the DNA barcode analysis of more than half of the recorded species. The checklist includes numerous new faunistic records for Crete and/or Greece, while also necessitating multiple corrections. Notably, nearly 10% of the fauna lacks a clear taxonomic assignment, highlighting an urgent need for integrative taxonomic research.

## 1. Introduction

Crete, with an area of 8261 km^2^, is the largest Greek island, located in the eastern Mediterranean Sea, south of the mainland. Crete’s topography is marked by mountainous landscapes, with three notable mountain ranges exceeding 2000 m: the Lefka Ori (White Mountains), Idi (Psiloritis), and Dikti. These ranges significantly impact the island’s Mediterranean climate, creating varied precipitation levels across Crete. Aridity levels generally increase from west to east and from north to south across the island [1]. The annual precipitation varies from 240 mm in the southeastern region to 2000 mm in the White Mountains. The temperature typically drops 6 °C per 1000 m. Above 1600 m, the precipitation is mostly snow, which accumulates from late October to May, sometimes extending to July in Lefka Ori [2]. Crete’s high mountains, mainly composed of limestone, surround the main island. In addition, extensive agricultural land and intensive touristic utilization of coastal areas are particularly prominent along the northern shoreline, whereas the southern regions remain comparatively undisturbed and close to their natural state. The coast also has 36 offshore islets, varying in geology and drier than the mainland.

**Figure 1:**
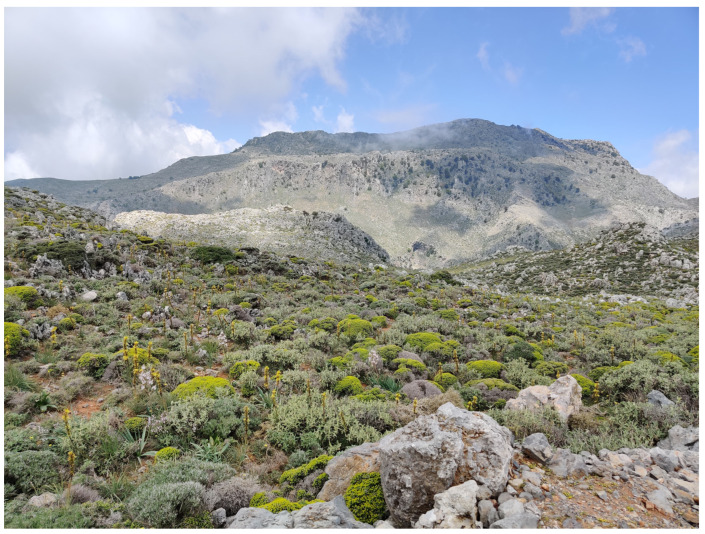
Crete is characterized by mountain habitats (Kapsodasos) (Photo P. Huemer).

**Figure 2:**
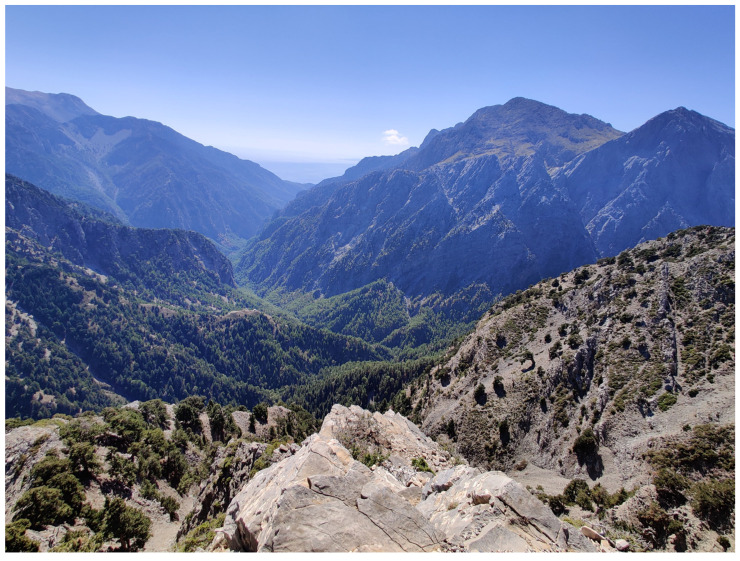
Extensive forest habitats still occur in the steep gorges (entrance to Samaria) (Photo P. Huemer).

**Figure 3:**
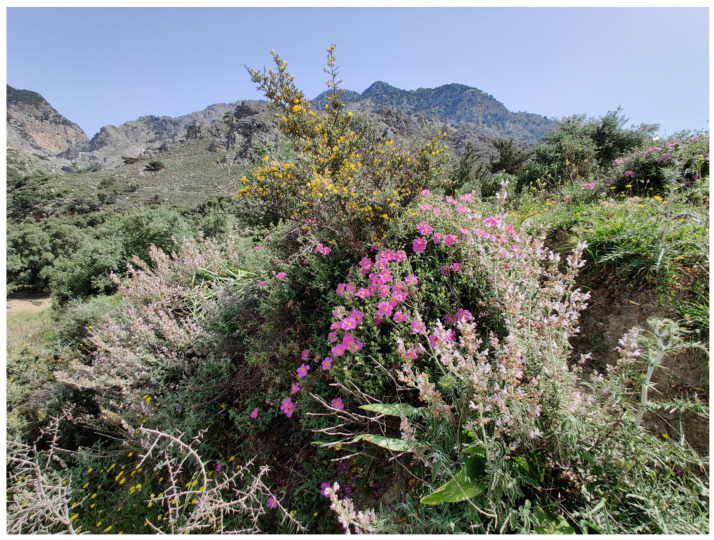
Rich maquis vegetation is widespread (near Zaros) (Photo P. Huemer).

**Figure 4:**
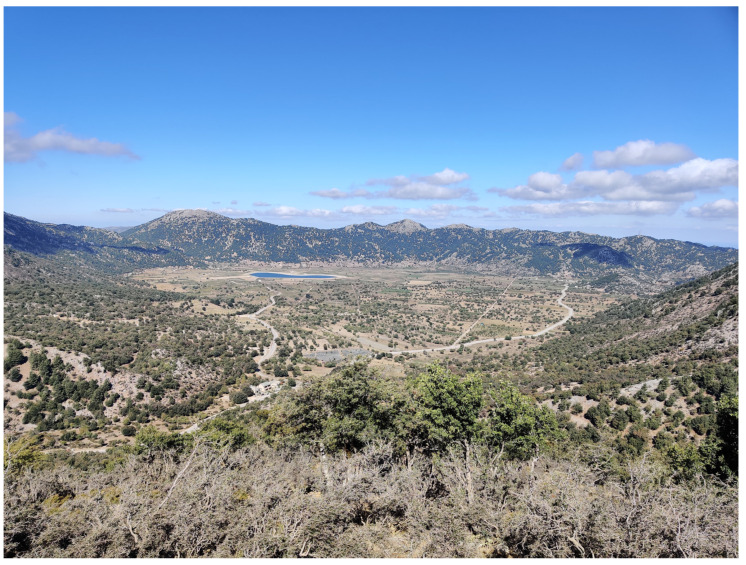
Agricultural influence is dominating easily accessible areas (Omalos Plateau) (Photo P. Huemer).

The Cretan landscape is dominated by “phrygana” (sensu [3]), “maquis” (mainly *Quercus coccifera*), and intermediate mosaic formations, in addition to subalpine shrubs. Phrygana comprises spiny, aromatic shrubs with brief lifespans, superficial roots, limited palatability, and resilience to drought and grazing. Examples include *Calicotome villosa*, *Sarcopoterium spinosum*, *Thymus capitatus*, and *Genista acanthoclada*. Cypress (*Cupressus sempervirens*), pines (*Pinus brutia*), and oak forests (*Quercus coccifera*, *Q. ilex*) also exist, mainly on mountain cliffs (Figure 1, Figure 2, Figure 3 and Figure 4). Lowland shrublands reach the alpine zones, confirming the weak zonation of Crete’s vegetation [4]. Crete’s unique orography and its long-standing isolation from the mainland—during glacial maxima, the sea level was 200 m lower than at present [5], yet Crete remained isolated from the Cyclades and Peloponnese, as the Cretan Sea is much deeper [6]—have contributed to an impressive level of endemism. This is especially well documented in vascular plants, with 223 endemics, encompassing about 10% of the entire flora [7,8], but also in many other groups of organisms, including even vertebrates such as *Crocidura zimmermanni* and several extinct species from the Holocene [6].

The first studies on the Lepidoptera fauna of Crete occurred in the mid-19th century [9,10]. However, intensive surveys were only conducted by Hans Rebel (1861–1940), culminating in 1916 with the first comprehensive faunal compilation and analysis, which identified 327 species [11]. It was already known at the time that this only represented a fraction of its actual species diversity. Eighty years later, the first modern catalog of European Lepidoptera more than doubled the number of recorded species, to 724 [12]. Since then, few comprehensive studies have focused explicitly on a broader spectrum of species from the island, or on specific families [10,13,14,15,16]. Numerous scattered reports of additional or newly described species have gradually enriched the known species inventory [17,18,19,20,21,22,23,24,25]. Taking these supplementary findings into account, 972 species were reported to be present [26].

Recently, macrophotography has played an increasingly important role in the collection of data on European Lepidoptera. The “Butterflies of Crete” project was specifically developed for Crete [27]. The site lists 45 butterfly species established on Crete, plus two regular immigrants (*Vanessa cardui* and *Danaus chrysippus*). The grid distribution maps for these species are quite convincing (e.g., https://butterfliesofcrete.com/family-nymphalidae/coenonympha-thyrsis/ accessed on 22 March 2025). One curious aspect is the list of 43 “missing butterflies of Crete” (https://butterfliesofcrete.com/family-hesperiidae/missing-butterflies-of-crete/) (accessed on 1 March 2025). These are species that were once reported by someone from Crete, but in the vast majority of cases, they can easily be explained by misidentifications or, in some instances, pure fantasy. However, the project also includes all other species of Lepidoptera ). As stated on the site: “Moths of Crete—Species—More than 1300 moth species are found on the island of Crete, inhabiting a wide range of habitats. There are thought to be approximately 160,000 moth species worldwide, many of which have yet to be described” [28]. The possibility that some of these species may also occur on Crete is not mentioned. Furthermore, the list of the alleged 1300 species is not provided anywhere. What is certain is that no validated or corrected list exists. There is, however, a photo list—although it is unclear whether it has been verified: “More photographs and detailed information on 600+ moth species on the island of Crete are available on the species pages; these are listed alphabetically (A–Z)”. If one follows the link, 617 species of moths are shown in photos (as of 21 March 2025), including 29 “new” species discovered in February and March 2025 alone. The photo project associated with iNaturalist continues to grow rapidly. In general, the identifications here are correct—or at least, they seem to be. However, this photo-based project has a major limitation: much of the information remains speculative, and cryptic species will never be discovered in this way. Without DNA barcoding, the only *Adela* species on Crete would always be *Adela paludicolella* (and not *Adela orientella*). Similarly, *Rivula tanitalis* could continue to be misidentified as *Rivula sericealis*, a species that does not even exist on Crete. The project assumes that (at least almost) all species of Crete are known, and that (almost) all species can be identified from photographs. Unfortunately—as shown here—neither assumption is entirely accurate. Nevertheless, this project has its merits and contributes to a better understanding of the Lepidoptera of Crete.

Despite the ongoing inventory of species on Crete, the assessment of genetic diversity through DNA barcoding has been restricted to selected species within the framework of broader studies, such as those on European butterflies, Gelechiidae, and Gracillariidae [29,30,31]. In recent years, however, molecular methods have also been applied to delineate cryptic species from Crete, including several newly described taxa [17,18,19,20,21,22,23,24,25]. Because the genetic assessment of Lepidoptera in the eastern Mediterranean—and for Crete in particular—is best described as highly fragmented, the primary goal of this study was to achieve the most comprehensive possible coverage of the island’s fauna through DNA barcoding [32]. The results serve as a basis for evaluating the uniqueness of the Cretan fauna, and to facilitate the generation of an updated checklist.

## 2. Materials and Methods

### 2.1. DNA Barcoding

**Figure 5:**
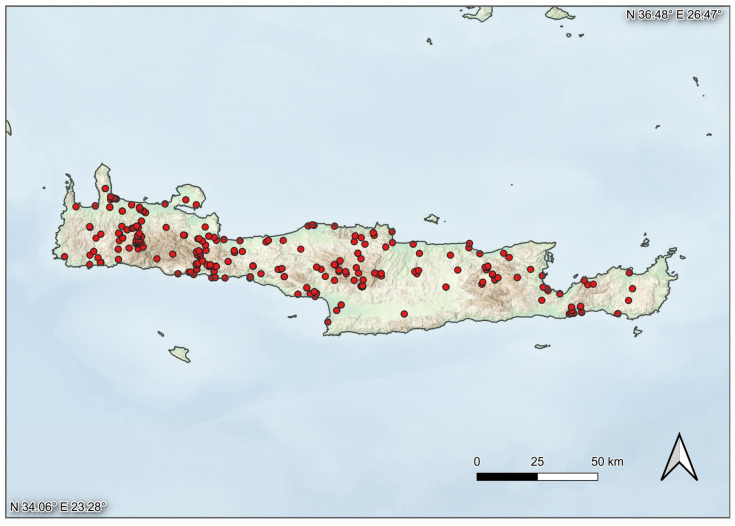
Localities of DNA-barcoded samples. OpenStreetMap: https://www.openstreetmap.org/copyright (accessed on 22 March 2025) CC BY-SA; OpenTopo: https://opentopomap.org/about#verwendung (accessed on 22 March 2025) CC BY-SA; SRTM: https://www.earthdata.nasa.gov/data/instruments/srtm (accessed on 22 March 2025) Public domain.

This study encompasses all Lepidoptera specimens from Crete that were accessible to us, including those from various external sources. However, it primarily relies on material collected by some of the authors—particularly Kai Berggren, Leif Aarvik, and Peter Huemer—during recent, privately funded, and independent expeditions, some lasting for several weeks. The main goal was to achieve the most comprehensive molecular analysis of the Lepidoptera fauna. The sampling program was carried out in targeted but geographically and seasonally representative cycles. Furthermore, different collection methods were employed to capture the most species: notably, light trapping during the night, and netting during the day and twilight. This approach, combined with the diverse taxonomic expertise of the specialists involved, allowed for the coverage of an unusually broad taxonomic spectrum, with full geographic coverage of the island (Figure 5). The freshly collected material was, for the most part, immediately pinned, spread, and dried on site. In some cases, genital dissections were performed at a later stage [33]. The selection of specimens for DNA barcoding was primarily based on morphological criteria such as wing patterns and coloration, head characteristics, and occasionally after preliminary examination of genital morphology. In addition to our own samples, material from Walter Ruckdeschel’s collection, who studied the island’s fauna intensively for nearly two decades, was analyzed and partially sequenced [14,15].

Tissue samples (dried legs) from 1712 specimens from the TLMF (Tyrolean Federal State Museums), provisionally identified to the morphospecies level, were prepared according to prescribed standards to obtain DNA barcode sequences of the mitochondrial COI gene (cytochrome c oxidase 1). The material was processed at the Canadian Centre for DNA Barcoding (CCDB, Centre for Biodiversity Genomics, University of Guelph, Guelph, ON, Canada), using a standard high-throughput protocol [34]. Additionally, 894 specimens from the Kai Berggren collection and 461 samples from the Zoological State Collection Munich were sequenced. Material from the Berggren collection was processed as part of the project Biodiversity Genomics Europe (BGE) at the University of Florence (Italy), where DNA was extracted using a non-destructive protocol. COI amplicons were generated through a single-step PCR using a combination of Folmer (LCO1490, HC02198; [35]) and Lep (LepF1, LepR1; [36]) primers, and sequencing was performed on an 8M ZMW SMRT cell on a PacBio Sequel IIe platform. Raw reads were demultiplexed using the Pacific Biosciences SMRT Link software https://www.pacb.com/smrt-link/ (accessed on 1 October 2024). Consensus sequences were generated with the PacBio Amplicon Analysis (pbaa) https://github.com/PacificBiosciences/pbAA (accessed on 1 October 2024) tool. Primer trimming, translation, and stop codon checking were performed using Geneious Prime 2024.0.1, while taxonomic validation was performed via BLAST (NCBI BLAST+ v2.14.0) against the NCBI nucleotide database [37].

Details including complete voucher data and images can be accessed in the public dataset “Lepidoptera of Crete” at dx.doi.org/10.5883/DS-LEPICRET in the Barcode of Life Database (BOLD) [38]. The remarkably extensive taxonomic coverage of this barcode library is also largely due to the inclusion of sequences for groups such as butterflies, Sesiidae, and some Microlepidoptera, which are already publicly available on BOLD.

All sequences were assigned to Barcode Index Numbers (BINs), an algorithm-based approach to delineate operational taxonomic units that provide a good proxy for species—which were automatically calculated for records in BOLD that were compliant with the DNA barcode standard [39]. A few BINs included specimens belonging to more than one taxon because of BIN sharing, misidentifications, or contaminations. Identification was based on external morphology and, in critical cases, on genitalia morphology. In case of BINs attributed to a single Linnean name, these were accepted as correct, although potential misidentifications cannot be fully ruled out.

### 2.2. Checklist

The checklist for the Lepidoptera of Crete presented in this study is primarily based on knowledge already published online on Lepiforum and follows the systematics and nomenclature used therein [26]. We omit the listing of synonyms. In a further step, additional verified species from various sources were included:

- Genetically validated additional species (some morphologically examined);
- Unpublished, morphologically verified specimens from collections.

Other species considered probable but not yet confirmed were included from the following sources:

- iNaturalist [40], including the “Butterflies of Crete” project [27,28];
- observation.org [41];
- Fauna Europaea [42].

However, the checklist does not contain species that are currently only identifiable to the genus level (Table S1). Finally, incorrect records, as well as unverified and unlikely records, mostly based only on photographs, were not included in the checklist.

## 3. Results

### 3.1. DNA Barcodes—General Overview

The sequence analysis of tissue samples from 3363 specimens generated 3110 DNA barcode sequences. Full DNA barcodes (658 bp) were recovered for 1792 specimens, while another 1238 specimens produced a sequence ranging from 600 to 657 bp. Only 80 specimens had a sequence of less than 600 bp. The mean intraspecific sequence divergence was 0.48%, while congeneric species possessed an average of 8.88% divergence.

### 3.2. BINs Attributed to Linnaean Names

BOLD analytics assigned a species-level identification to 724 Linnaean names, and 719 species were automatically assigned to a species based on their BIN on the BOLD System. Among these, members of 21 species were assigned to two BINs, while members of 5 species were placed in three BINs, based solely on our sequences from Crete. The evidence for these splits is available in Table S2 and in the BOLD dataset.

These 26 species show moderate genetic variability on Crete, up to about 2%, and merit further in-depth morphological analysis. For example, *Gelechia senticetella* may comprise 2–3 distinct species. Specimens of *Maniola jurtina* also exhibit considerable genetic diversity, as they were assigned to three BINs, one of which matches *Maniola telmessia* (Zeller, 1847), a species known from the eastern Aegean.

**Figure 6:**
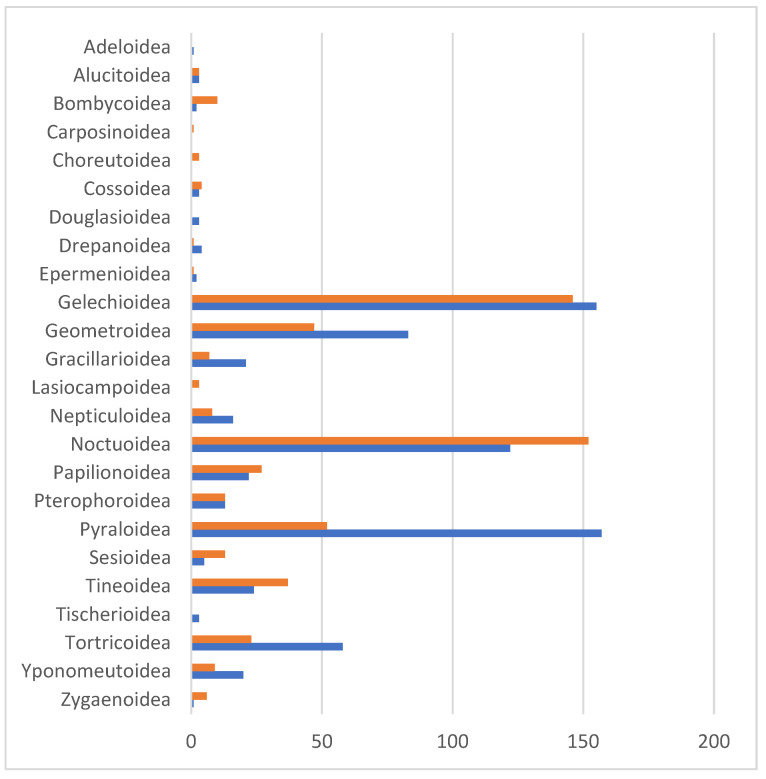
Species numbers per superfamily from Crete, with BIN (blue) or without BIN (orange). Unassigned BINs were not considered.

These BINs belonged to 21 of the 24 superfamilies from Crete (Figure 6). The Pyraloidea, Gelechioidea, Noctuoidea, and Geometroidea not only have the highest species numbers but also show particularly good BIN coverage. Currently, no BINs are available for three species-poor superfamilies (Carposinoidea, Choreutoidea, Lasiocampoidea).

BIN sharing was only detected in eight closely related species pairs for *Agonopterix straminella*/*A. scopariella*, *Cnephasia genitalana*/*C. longana*, *Pelochrista caecimaculana*/*P. modicana*, *Dioryctria mendacella*/*D. pineae*, *Cadra delatinella*/*C. furcatella*, *Camptogramma bilineata*/*C. grisescens*, *Scopula decolor*/*S. imitaria*, and *Euxoa temera*/*E. distinguenda*. Five species with shorter sequences did not qualify for a BIN assignment, while no specimens of 476 species were available for analysis.

### 3.3. Unassigned BINs—Potential Cryptic Diversity

Another 112 BINs could not be assigned to a Linnaean species based on the reference sequences available in BOLD; 109 of these BINs could be assigned to a genus via BOLD, while 3 could only be assigned to a family (exclusively Tineidae). From this total, 76 are only known from Crete, whereas the other 36 BINs have been recorded elsewhere. Additionally, two distinct sequences restricted to Crete, which lack BINs, could not be assigned to any species (Table S1).

The BINs lacking a species assignment likely belong either to already known but insufficiently revised species complexes, or to groups that have seen little barcode analysis. This latter situation applies, for example, to the Autostichidae, which have few barcode records from Europe. A potentially larger number of unidentified BINs suggests previously unknown intraspecific divergences. This is likely to include some of the 24 species with a distance of less than 2% to their nearest neighbor. Conversely, 36 species show divergences of 4–11% from their closest relative in BOLD, where the largest proportion of additional and potentially even undescribed species is likely to be found.

**Figure 7:**
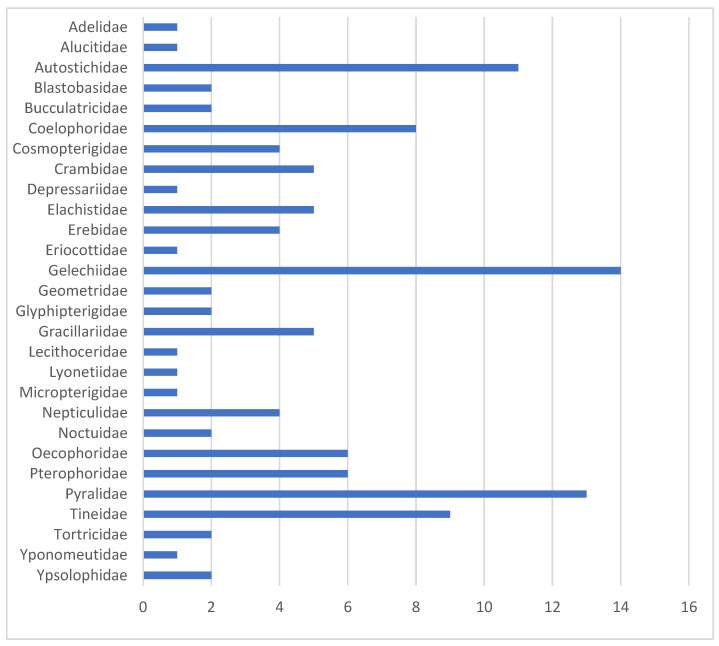
Number of unassigned BINs per family.

The unassigned BINs are distributed very unevenly across major taxonomic groups (Figure 7). Among the 27 families with unknown BINs, the Gelechiidae are particularly well represented, with 14 BINs, followed by the Pyralidae with 13 BINs and the Autostichidae with 11 BINs. Additionally, Pterophoridae, Tineidae, and Coleophoridae each contain more than five BINs lacking a species assignment. In seven predominantly species-poor families, only a single BIN that could not be classified at the species level was recorded. For all of these taxa, extensive morphological studies are required to ultimately assign BINs to Linnaean names.

### 3.4. New Faunistic Records

#### 3.4.1. New Records with Accompanying DNA Barcodes

A total of 125 species from 30 families represented new records for Crete, based on barcode recovery and unequivocal assignment to a Linnaean taxon (Table 1). Some of these species were initially identified based on morphological characteristics and subsequently confirmed through DNA barcoding. Occasionally, records have already been reported in Lepiforum (e.g., *Mondeguina mediterranella*), while others have been published without reliable evidence (e.g., *Elachista scirpi*) [10]. Detailed information on the collection circumstances and sequences can be found in the dataset “Lepidoptera of Crete” at dx.doi.org/10.5883/DS-LEPICRET. For some of these species, there is additional unsequenced voucher material, and in some cases, photographs are available on online platforms.

The list of new discoveries also includes 36 faunistically remarkable first records for Greece, and even for Europe, including *Ostrinia furnacalis* and *Agriphila bleszynskiella*.

#### 3.4.2. New Records Based on Morphology

Twenty-three species from 10 families were newly recorded from Crete, based solely on morphological confirmation, and were identified either phenotypically and/or through genitalia preparations (Table 2; for label data, see Table S3).

#### 3.4.3. Reliable New Records Based on Photographs

The presence of another 16 species in Crete is so far based solely on photographs (Table 3). Of course, it is always possible that apparently unmistakable species (e.g., *Mimas tiliae*, *Noctua interjecta*, *Apocheima hispidaria*) have their own island endemics. There is a nice photo sequence of *Hemaris croatica* visiting a flower, but since this conspicuous species has not been recorded elsewhere on Crete, it is probably a rare immigrant without a self-sustaining population. There is only a single caterpillar photo of *Euproctis chrysorrhoea*, so it is unclear whether it is established on Crete. The Australian Crambidae *Heliothela ophideresana* was known from Africa and Iran as well, but not yet from Europe. A nice photo sequence from Crete is told to show this species [28]—a DNA barcode would help to confirm this identification.

### 3.5. Revised and Updated Checklist

The revised checklist for the Lepidoptera of Crete is based on the taxonomy and nomenclature adopted by Lepiforum from various sources [26]. Important references to records are included, without claiming completeness, especially for genetically unverified species. The checklist currently includes 1230 confirmed species belonging to 62 families (Table S2). However, it should be noted that a considerable number of taxa that have not been genetically examined may still pose taxonomic issues in the future.

Our list excludes 212 species previously reported from Crete. Of these, 54 species represent misidentifications, while 83 species remain uncertain—either because they are only documented through photos that do not allow for reliable identification, or because the literature sources do not align with the species’ known distribution patterns and material was not available to us. A special case involves 43 species that were previously listed as part of Crete’s fauna, but for which no concrete evidence of occurrence exists [42]; most of these were likely included based on the assumption that they should be present. The same is the case for 32 species of butterflies, listed as “missing species” [27]. However, it is possible that a few of the excluded species may be present (Table S4).

If the 112 BINs that have not yet been identified to the species level are included, the actual number of species could reach approximately 1400. However, the verification of these taxa is reserved for future studies, some of which are already underway.

## 4. Discussion

Crete has long been a focus of research on lepidopterans, and it is therefore considered to be both extensively studied and well sampled. However, the assessment of genetic diversity has largely been neglected to date. Aside from a few recent descriptions, gene sequences—particularly DNA barcodes—have only been published or made publicly available for very few species-rich groups [29,30,31]. The barcode library assembled in this study provides records for more than half of the known fauna, with 724 sequenced and reliably identifiable species (719 with BINs). Such a comprehensive genetic survey has not yet been recorded for any Mediterranean island.

**Figure 8:**
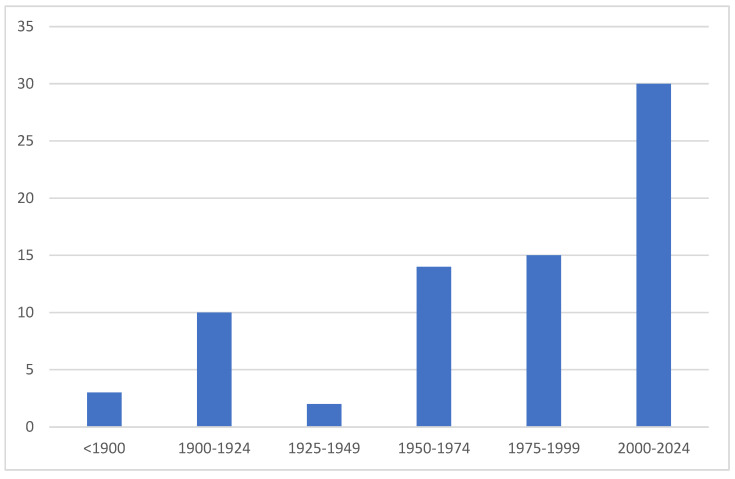
Number of endemic species described per period.

The clearly insufficient taxonomic study of Crete’s fauna is reflected, among other aspects, by the documentation of endemic and, thus, particularly significant species (Figure 8). Of the 75 known species, 40% have been described after 2000. Additional species, such as the recently discovered *Melathrix edmundsi* and *Leucochlaena labrys*, suggest that more unrecognized endemics await taxonomic clarification [43,44]. Moreover, based on current knowledge, it is very likely that numerous other endemics are among the still-unresolved species. The presence of more than 100 BINs that lack a species assignment is unusually high for a region considered to be well researched. Certain families, such as Gelechiidae and Gracillariidae, include many unresolved species, considering an already largely complete European DNA barcoding library and the high number of unknown BINs [30,31]. Similarly, initial research suggests that even less diverse families, such as Cosmopterigidae, may contain three undescribed species within the genera *Pyroderces*, *Sorhagenia*, and *Alloclita*. Additionally, three species of the family Erebidae, represented by the genera *Hypenodes* and *Orectis*, require taxonomic revision in the former genus or are likely new to science in the latter genus. A similar situation applies to several representatives of Pyraloidea. However, all of these taxa require comprehensive taxonomic analyses, including type studies. In other families with more unknown BINs, such as Autostichidae, Pterophoridae, Coleophoridae, Elachistidae, or Oecophoridae, the lack of reference sequences is likely responsible for the current classification deficits, and even some new species may be involved.

Our study not only addresses a significant gap in the genetic documentation of biodiversity in Crete but also highlights the considerable gaps in knowledge of the island’s fauna. The last comprehensive publication with a checklist included just over 700 species [12], while approximately 300 additional species records are reported in various online media. The present faunistic–taxonomic surveys have significantly expanded knowledge of the fauna, including 125 newly reported and genetically confirmed species, as well as 23 additional species based solely on morphology. More discoveries are certain, as the areas above 1600 m have hardly been studied

At the same time, molecular verification of previously published taxa allowed for the correction of numerous misidentifications. Finally, 212 species, either officially published or shared in online forums, had to be eliminated as invalid or doubtful records. All of these findings can be considered for a completely revised and updated checklist of Crete’s fauna.

Finally, even after a thorough revision of currently unresolved species, it is not yet possible to make a definitive assessment of the lepidopteran fauna of Crete. This will only become feasible through planned expansions of sequencing efforts to include the remaining species, which are highly likely to yield further surprises.

## 5. Conclusions

Genetically substantiated species inventories are currently lacking for all other major Mediterranean islands. However, the results from Crete demonstrate the need to prioritize such inventories, particularly on islands with high endemic potential. As evidenced in Crete, the sequence analysis of species whose taxonomic status seems unequivocal often reveals cryptic diversity and previously unrecognized endemism [15,16,17,22]. Therefore, the goal of faunistic surveys, particularly in the less explored Mediterranean islands, should involve the comprehensive analysis of all morphospecies using DNA barcodes, followed by the application of integrative taxonomic methods to further investigate taxa showing genetic divergence.

## Figures and Tables

**Table 1:** New faunistic records for Crete with DNA barcodes; * new record for Greece; ** new record for Europe.

Alucitidae	Nepticulidae
*Alucita grammodactyla* (Zeller, 1841) *	*Stigmella basiguttella* (Heinemann, 1862)
Bedelliidae	*Stigmella fasciata* (van Nieukerken and Johansson, 2003)
*Bedellia ehikella* (Szőcs, 1967)	*Trifurcula eurema* (Tutt, 1899)
Blastobasidae	Noctuidae
*Blastobasis glandulella* (Riley, 1871) *	*Bryophila felina* (Eversmann, 1852)
*Tecmerium perplexus* (Gozmány, 1957)	*Caradrina kadenii* Freyer, [1836]
Bucculatricidae	Nolidae
*Bucculatrix bechsteinella* (Bechstein and Scharfenberg, 1805) *	*Meganola kolbi* (Daniel, 1935)
*Bucculatrix phagnalella* (Walsingham, 1908) *	*Nola subchlamydula* (Staudinger, 1870)
Coleophoridae	Oecophoridae
*Coleophora berbera* (Baldizzone, 1988) *	*Batia lunaris* (Haworth, 1828)
*Coleophora curictae* (Baldizzone, 2016) *	*Batia samosella* (Sutter, 2003)
*Coleophora deauratella* (Lienig and Zeller, 1846)	Plutellidae
*Coleophora nutantella* (Mühlig and Frey, 1857)	*Rhigognostis annulatella* (Curtis, 1832)
*Coleophora oriolella* (Zeller, 1849)	Pyralidae
*Coleophora variicornis* (Toll, 1952)	*Acrobasis advenella* (Zincken, 1818)
Cosmopterigidae	*Acrobasis centunculella* (Mann, 1859)
*Anatrachyntis badia* (Hodges, 1962)	*Acrobasis consociella* (Hübner, [1813])
Crambidae	*Acrobasis getuliella* (Zerny, 1914) *
*Agriphila bleszynskiella* (Amsel, 1961) **	*Acrobasis glaucella* (Staudinger, 1859)
*Cataclysta lemnata* (Linnaeus, 1758)	*Acrobasis marmorea* (Haworth, 1811)
*Euchromius ramburiellus* (Duponchel, 1836)	*Acrobasis obtusella* (Hübner, 1796)
*Euchromius vinculellus* (Zeller, 1847)	*Amphithrix sublineatella* (Staudinger, 1859) *
*Metasia carnealis* (Treitschke, 1829)	*Coenochroa ablutella* (Zeller, 1839)
*Pediasia contaminella* (Hübner, 1796)	*Dioryctria mendacella* (Staudinger, 1859)
*Ostrinia furnacalis* (Guenée, 1854) **	*Dioryctria pineae* (Staudinger, 1859)
Depressariidae	*Elegia atrifasciella* (Ragonot, 1887)
*Agonopterix nodiflorella* (Millière, 1866)	*Ephestia cypriusella* (Roesler, 1965)
*Agonopterix straminella* (Staudinger, 1859)	*Epischnia asteris* (Staudinger, 1870) *
*Depressaria bantiella* (Rocci, 1934) *	*Epischnia cretaciella* (Mann, 1869)
*Depressaria discipunctella* Herrich-Schäffer, [1854]	*Etiella zinckenella* (Treitschke, 1832)
*Rosetea corfuella* (Lvovsky, 2000)	*Euzophera bigella* (Zeller, 1848)
Douglasiidae	*Euzophera lunulella* (Costa, [1836])
*Tinagma ocnerostomella* (Stainton, 1850)	*Euzophera osseatella* (Treitschke, 1832)
Drepanidae	*Euzopherodes vapidella* (Mann, 1857)
*Cilix glaucata* (Scopoli, 1763)	*Keradere tengstroemiella* (Erschoff, 1874)
Elachistidae	*Melathrix coenulentella* (Zeller, 1846) *
*Blastodacna atra* (Haworth, 1828)	*Merulempista brucella* (Staudinger, 1879)
*Elachista differens* (Parenti, 1978)	*Merulempista turturella* (Zeller, 1848) *
*Elachista gleichenella* (Fabricius, 1781)	*Morosaphycita cleopatrella* (Ragonot, 1887) *
*Elachista pigerella* (Herrich-Schäffer, [1854])	*Myelois ossicolor* (Ragonot, 1893) *
*Elachista scirpi* (Stainton, 1887)	*Nyctegretis ruminella* (De la Harpe, 1860)
*Perittia echiella* (De Joannis, 1902) *	*Pempelia brephiella* (Staudinger, 1879) *
Epermeniidae	*Phycita acericola* (Kuznetsov, 1960)
*Epermenia chaerophyllella* (Goeze, 1783)	*Phycita torrenti* (Agenjo, 1962)
Erebidae	*Phycitodes inquinatella* (Ragonot, 1887)
*Eilema muscula* (Staudinger, 1899)	*Phycitodes lacteella* (Rothschild, 1915)
Gelechiidae	*Phycitodes saxicola* (Vaughan, 1870)
*Anarsia leberonella* (Réal, 1994)	*Psorosa mediterranella* (Amsel, 1953) *
*Aproaerema montanata* (Gozmány, 1957)*	*Pyralis kacheticalis* (Christoph, 1893)
*Aristotelia subdecurtella* (Stainton, 1859)	*Rhodophaea formosa* (Haworth, 1811) *
*Bryotropha figulella* (Staudinger, 1859)	Scythropiidae
*Carpatolechia decorella* (Haworth, 1812)	*Scythropia crataegella* (Linnaeus, 1767)
*Mondeguina mediterranella* (Nel and Varenne, 2012) *	Tineidae
*Monochroa hornigi* (Staudinger, 1883) *	*Nemapogon hungaricus* (Gozmány, 1960)
*Oxypteryx immaculatella* (Douglas, 1850) *	*Opogona omoscopa* (Meyrick, 1893) *
*Parastenolechia nigrinotella* (Zeller, 1847) *	Tischeriidae
Geometridae	*Coptotriche angusticolella* (Duponchel, [1843])
*Chloroclysta siterata* (Hufnagel, 1767)	Tortricidae
Glyphipterigidae	*Bactra lancealana* (Hübner, [1799]) *
*Acrolepiopsis vesperella* (Zeller, 1850)	*Cnephasia genitalana* (Pierce and Metcalfe, 1915) *
*Orthotelia sparganella* (Thunberg, 1788)	*Cydia conicolana* (Heylaerts, 1874) *
Gracillariidae	*Cydia johanssoni* (Aarvik and Karsholt, 1993)
*Acrocercops brongniardella* (Fabricius, 1798)	*Cydia splendana* (Hübner, [1799])
*Acrocercops tacita* (Triberti, 2001)	*Epiblema cirsiana* (Zeller, 1843) *
*Aspilapteryx tringipennella* (Zeller, 1839)	*Epiblema cnicicolana* (Zeller, 1847) *
*Calybites phasianipennella* (Hübner, [1813])	*Notocelia cynosbatella* (Linnaeus, 1758)
*Parornix anguliferella* (Zeller, 1847)	*Pammene blockiana* (Herrich-Schäffer, 1851) *
*Parornix tenella* (Rebel, 1919) *	*Pammene gallicolana* (Lienig and Zeller, 1846)
*Phyllonorycter cephalariae* (Lhomme, 1934)	*Pelochrista caecimaculana* (Hübner, [1799]) *
*Phyllonorycter corylifoliella* (Hübner, 1796)	*Pelochrista modicana* (Zeller, 1847)
*Phyllonorycter distentella* (Zeller, 1846) *	*Phtheochroa ochralana* (Chrétien, 1915)
*Phyllonorycter triflorella* (de Peyerimhoff, 1871)	*Zeiraphera isertana* (Fabricius, 1794) *
*Triberta cistifoliella* (Groschke, 1944)	Yponomeutidae
Momphidae	*Paradoxus osyridellus* (Millière, 1869)
*Mompha epilobiella* ([Denis and Schiffermüller], 1775)	Ypsolophidae
	*Ypsolopha alpella* ([Denis and Schiffermüller], 1775)

**Table 2:** New faunistic records from Crete, based solely on morphology.

Taxon	Family
*Coleophora kroneella* (Fuchs, 1899)	Coleophoridae
*Coleophora nigridorsella* (Amsel, 1935)	Coleophoridae
*Coleophora ptarmicia* (Walsingham, 1910)	Coleophoridae
*Phlyctaenomorpha sinuosalis* (Le Cerf, [1910])	Crambidae
*Tegostoma comparalis* (Hübner, 1796)	Crambidae
*Depressaria depressana* (Fabricius, 1775)	Depressariidae
*Eublemma purpurina* ([Denis and Schiffermüller], 1775)	Erebidae
*Caryocolum mucronatella* (Chrétien, 1900)	Gelechiidae
*Lanceoptera panochra* (Janse, 1960)	Gelechiidae
*Phyllonorycter platani* (Staudinger, 1870)	Gracillariidae
*Phyllonorycter sublautella* (Stainton, 1869)	Gracillariidae
*Mompha divisella* Herrich-Schäffer, [1854]	Momphidae
*Stigmella muricatella* (Klimesch, 1978)	Nepticulidae
*Mesapamea secalis* (Linnaeus, 1758)	Noctuidae
*Meganola kolbi* (Daniel, 1935)	Nolidae
*Meganola togatulalis* ([Denis and Schiffermüller], 1775)	Nolidae
*Pyla fusca* (Haworth, 1811)	Pyralidae
*Nemapogon variatella* (Clemens, 1859)	Tineidae
*Aethes bilbaensis* (Rössler, 1877)	Tortricidae
*Cnephasia ecullyana* Réal, 1951	Tortricidae
*Epinotia festivana* (Hübner, [1799])	Tortricidae
*Notocelia mediterranea* (Obraztsov, 1952)	Tortricidae
*Rhyacionia buoliana* ([Denis and Schiffermüller], 1775)	Tortricidae

**Table 3:** New faunistic records from Crete, based solely on photographs.

Taxon	Family
*Heliothela ophideresana* (Walker, 1863)	Crambidae
*Palepicorsia ustrinalis* (Christoph, 1877)	Crambidae
*Pyrausta purpuralis* (Linnaeus, 1758)	Crambidae
*Acantholipes regularis* (Hübner, 1813)	Erebidae
*Euproctis chrysorrhoea* (Linnaeus, 1758)	Erebidae
*Apocheima hispidaria* ([Denis and Schiffermüller], 1775)	Geometridae
*Agrotis herzogi* (Rebel, 1911)	Noctuidae
*Noctua interjecta* Hübner, [1803]	Noctuidae
*Aphomia cephalonica* (Stainton, 1866)	Pyralidae
*Denticera divisella* (Duponchel, [1843])	Pyralidae
*Galleria mellonella* (Linnaeus, 1758)	Pyralidae
*Myelois circumvoluta* (Geoffroy in Fourcroy, 1785)	Pyralidae
*Hemaris croatica* (Esper, 1800)	Sphingidae
*Mimas tiliae* (Linnaeus, 1758)	Sphingidae
*Cochylimorpha langeana* (Kalchberg, 1898)	Tortricidae
*Zelleria hepariella* (Stainton, 1849)	Yponomeutidae

## Data Availability

All 3110 COI sequences are available in the dataset “Lepidoptera of Crete” DS-LEPICRET on BOLD (dx.doi.org/10.5883/DS-LEPNCYPR), at https://www.boldsystems.org/ (accessed on 8 March 2025).

## Supplementary Materials

The following supporting information can be downloaded at https://www.mdpi.com/article/10.3390/insects16050438/s1: Table S1: Unidentified BINs; Table S2: Checklist of Lepidoptera from Crete; Table S3: Label data of new records for Crete based solely on morphology; Table S4: Incorrect or doubtful species records. References [45,46,47,48,49,50,51,52,53,54,55,56,57,58,59,60,61,62,63,64,65,66,67,68,69,70,71,72,73,74,75,76,77,78,79,80,81,82,83,84,85,86,87,88,89,90,91,92,93,94,95,96,97,98,99,100,101,102,103,104,105,106,107,108,109,110,111,112,113,114,115,116,117,118,119,120,121,122,123,124,125,126,127,128,129,130,131,132,133,134,135,136,137,138,139,140,141,142,143,144,145,146,147,148,149,150,151] are cited in the Supplementary Materials.
